# Implementation of a workplace protection system and its correlation with experiences of workplace violence: a cross-sectional study

**DOI:** 10.1186/s12889-023-16112-w

**Published:** 2023-06-27

**Authors:** Eun-Mi Baek, BoKim Lee

**Affiliations:** 1 Department of Preventive Medicine, College of Medicine, The Catholic University of Korea, 06591 Seoul, South Korea; 2 Department of Nursing, University of Ulsan, 93 Daehak-ro, Nam-gu, 44610 Ulsan, South Korea

**Keywords:** Service workers, Workplace violence, Service protection system

## Abstract

**Background:**

While customer interactions are inherent and essential aspects of the service industry, instances of violence against service workers have brought social attention to the need for a system to ensure their protection. In South Korea, a protection system for the health of service workers has been implemented to prevent this type of violence and its negative consequences. This study conducted a comparative analysis to clarify the impacts of this protection system across a sample of service workers. We collected data on their general characteristics, occupational characteristics, and experiences with the service protection system to determine how those factors were related to workplace violence, with a focus on whether the system has reduced such occurrences.

**Methods:**

We collected self-reported survey data over 28 days (March 2 to March 30, 2020), resulting in 1,349 (99.3%) responses for our final analysis. We conducted a chi-square test and logistic regression analysis to investigate the general and occupational characteristics, experiences of violence, and experiences with the worker protection system.

**Results:**

We found workplace violence is more observed among males, older workers, electronic equipment repairers, irregular workers, and those who worked for extended periods. On the other hand, we found a reduction in the occurrence of workplace violence in businesses that provided service workers with regular counseling from professional counselors, had designated persons responsible for grievance procedures, and/or had grievance procedure committees. We found the lowest likelihood of workplace violence in businesses that operated stress relaxation programs (all *p* < 0.01).

**Conclusions:**

This study identified a correlation between the adoption of the protection system for service workers and the prevalence of workplace violence. We also clarified the effects of the service protection system and developed a plan for its expansion.

**Key points:**

This study clarified the correlation between the adoption of the protection system for service workers and the occurrence of workplace violence. Along with our investigation of the protection system’s effects, these findings provide a basis for expanding Korea’s existing worker protection system.

## Background

Industrial structures are changing on a global scale. Given the widespread shift from a manufacturing-centered to service-centered society, the number of service workers is steadily increasing. This is particularly evident in some countries. According to the Ministry of Employment and Labor, the South Korean service workforce contained approximately 11.28 million persons in 2018, which is an increase of around 244% since 1998; comparing industries, this is much higher than the 45.2% increase seen in the manufacturing workforce over the same period [1, 2].

Face-to-face interactions with clients are essential aspects of the service industry, but a recent increase in the rate of violence against service workers has highlighted the need to ensure their protection; thus, various methods are being explored [3–5]. Workplace violence (WV) is understood as any type of act, incident, or behavior in which a person is abused, threatened, humiliated, or assaulted in the workplace, including verbal and physical assaults [3].

More specifically, workplace violence and harassment refer to a range of unacceptable behaviors, practices, and threats that cause, aim to cause, or will likely result in physical, psychological, or economic harm [6].

While several studies have examined violence against service workers, the majority have focused on healthcare personnel; for example, one study found that 79.1% of participants had experienced physical violence, while others have reported verbal harassment rates of 40.2% [3, 4] and 22%-90% [7]. Still, it is clear that this type of violence frequently occurs elsewhere. Over the past year, 9.3% of salespeople at department stores and large discount stores have experienced physical violence [8]. A similar study found that travel industry workers frequently interacted with rude clients and experienced negative emotions [9]. Compared to the 6% rate of workplace violence experienced by general workers [7], it is clear that teachers and service workers face high exposure, and may thus experience negative health effects, including depression, insomnia, anxiety, exhaustion, and post-traumatic stress disorder [10]. The same problems are found in the home-visit context, where 50.3% of workers who experienced verbal abuse and 25.7% of those who experienced sexual harassment also developed health issues such as depression, insomnia, and exhaustion [10]. In sum, threats of workplace violence damage the quality of life for workers and their families by negatively impacting their physical health (pain, nervousness, headache, fatigue, and stomachache) and psychological health (depression, anxiety, and fear) [11, 12]. Many workers also use sick leave after exposure [13], which may lead to negative outcomes on organizational performance. This may emerge as declined job satisfaction, decreased workplace participation, and even increased turnover intention [14].

In South Korea, authorities have aimed to prevent violence against service workers and its negative outcomes through a provision in the Occupational Safety and Health Act of October 2018, therein requiring business owners to protect the health of their workers [15, 16]. This new regulation outlines multiple provisions, including preliminary actions to prevent health hazards caused by customer violence (e.g., phrases or audio guides for preventing verbal abuse, customer response preparation and training), necessary actions following violent incidents (e.g., work interruptions and phone calls, extended break times, treatment and counseling), and the legal prohibition of poor treatment toward workers. Moreover, the Korean government imposes fines and penalties on business owners who fail to comply with requirements that aim to increase the effectiveness of the act.

Despite these efforts, media reports occasionally describe instances in which the service worker protection system is violated; moreover, research has shown that it has not been fully implemented at all businesses [17]. Still, no previous scientific studies have demonstrated the effectiveness of the service worker protection system. To address this gap, the current study examined whether the human service protection system has effectively prevented workplace violence two years after its implementation based on actual occurrence rates. Specifically, we investigated (1) how different companies have implemented the safety protection system and (2) the correlation between the implementation approach and the level of workplace violence.

In this paper, we aim to provide a comprehensive understanding of the factors contributing to patient-induced violence and the organizational measures that can be taken to prevent it. To do so, we draw upon two key theoretical frameworks: Social Learning Theory and Contextual Theory.

Social Learning Theory, introduced by Bandura (1977), posits that individuals learn and acquire new behaviors through observation and imitation of others, particularly when these behaviors are reinforced by external factors. In the context of patient-induced violence, this theory suggests that exposure to aggressive behavior within healthcare settings may lead individuals to adopt and display violent tendencies themselves. It is essential to consider the role of social learning in understanding the perpetuation of violence within healthcare organizations.

Moreover, we turn to the Contextual Theory, as proposed by Johns (2006), to explore the prevention of violence at the organizational level. Johns emphasizes the significant impact of context on organizational behavior and proposes that contextual factors, such as organizational culture, climate, and leadership, can influence employees' actions and attitudes. In the case of patient-induced violence, this theory highlights the importance of understanding and addressing the contextual factors that may contribute to a hostile environment, thereby enabling organizations to develop and implement effective strategies to mitigate violence.

In summary, the Social Learning Theory and Contextual Theory together provide a robust theoretical foundation for our investigation into patient-induced violence and its prevention in healthcare settings. By considering the social learning processes that may perpetuate aggressive behavior, as well as the contextual factors that can influence organizational responses, we aim to develop a more nuanced understanding of the factors contributing to violence in healthcare settings and the potential interventions that may be employed to prevent it.

## Methods

We focused on three specific study purposes. First, we examined and compared experiences of workplace violence among participants based on their general and occupational characteristics. Second, we examined and compared experiences of workplace violence among participants according to their experiences with the service worker protection system. Third, we analyzed how the service worker protection system has affected workplace violence. Since South Korea has a more detailed and established service worker protection system relative to those used in other countries, our findings constitute valuable insights for practitioners and policymakers.

### Study design

In this cross-sectional survey study, we distributed self-reported questionnaires to a sample of service workers to collect data pertaining to the effects of the service worker protection system on workplace violence.

### Participants and data collection

Data were sampled from institutions recommended by the operators of the representative organization of Korean Labors. Based on the fact that these 7 organizations mostly experience violence from customers, we sampled from 7 organizations to improve the generality of this study. We applied the convenience sampling method. Convenience sampling is a non-probability sampling technique that involves selecting participants based on their accessibility and availability. The advantages of convenience sampling include its cost-effectiveness, time efficiency, and ease of implementation [18]. It is particularly useful when the research project has limited resources and time constraints, as it allows for quick recruitment of participants [19]. It was investigated that the workers of the 7 institutions we chose suffered a breach of work. Therefore, it was possible to secure data that could realize the purpose of this study using the convenience sampling method.

Service workers who had experienced workplace violence in various types of employment, including sales/business, call centers, electronic equipment repair, jobs with special classifications (e.g., Contract Labour), visiting work, staff at medical and social welfare facilities, and others (e.g., food and beverage workers, drivers, childcare workers, building managers, civil affairs workers, and firefighters). All participants understood our study purposes and objectives, were able to respond to the survey, and provided written consent to participate.

We collected data for a period of 28 days (March 2 to March 30, 2020) after receiving approval (MC21QISI0022) from the Institutional Review Board of Catholic University. The researchers explained the study to the administrators at each of the seven associations and organizations included in this study; we obtained written informed consent and collected data through the affiliates of the Federation of Democratic Trade Unions. As such, we distributed the questionnaires to workers in industries registered with the service union. The questionnaires took approximately 10 min complete and were collected immediately, then sealed in individual envelopes and delivered to the researchers. Thus, we received a total of 1,358 surveys; after excluding 10 that contained incomplete answers (0.7%), our final analysis included data from 1,349 (99.3%).

### Study tools

The questionnaires asked for general characteristics, including sex, age, average monthly income, occupation type, employment type, work experience, and weekly working hours, as well as information on six types of violence, including physical violence, verbal harassment, sexual harassment, intimidation or threats, unwanted sexual attention, and abusive behaviors. Workplace violence is described as any act or threat of physical violence, harassment, intimidation, or other threatening disruptive behavior that occurs at the work site. It ranges from threats and verbal abuse to physical assaults and even homicide [20]. The operational definition of workplace violence is experiencing "verbal abuse," "unwanted sexual attention," "threats," and "humiliating behavior" from individuals related to work, such as superiors, colleagues, subordinates, or customers, while performing work-related tasks.

In the study, experiences of violence were measured by asking the following questions: "Have you ever experienced verbal abuse related to work?", "Have you ever experienced unwanted sexual attention related to work?", "Have you ever experienced threats related to work?", and "Have you ever experienced humiliating behavior related to work?" The response options were scored as 1 point for "Yes, experienced within the last 1 month while performing work-related tasks" and 0 points for "No or Don't know." If the participant answered "Yes" to any of the questions, they were considered to have experienced workplace violence. We used violence tools from the sixth data wave of the 2017 Working Environment Survey. The workplace violence scale has a Cronbach’s ⍺ value of 0.80, indicating acceptable internal consistency [21]. Moreover, our measure of workplace violence was validated against a widely accepted tool, yielding a strong correlation (*r* = 0.85, *p* < 0.01), demonstrating its criterion validity [22].

In this study, we only analyzed incidences of violence that were directly experienced by the participants. From the workplace perspective, we examined the following preliminary action systems: (1) regular counseling for service workers by professional counselors, (2) the establishment of departments or individuals in charge of protecting service workers, (3) company policies to protect the health of service workers, (4) the designation of separate budgets for workers’ health problems, (5) company-conducted surveys on the state of emotional labor, types of customers, and health problems of workers, (6) authorization for workers to discontinue services in response to excessive demands (For example, stop phone service for abusive customers), (7) worker suggestion systems, (8) the designation of a person in charge of grievance procedures or a grievance procedure committee, (9) stress relief programs, (10) improvements in working conditions (e.g., the installation of rest facilities and welfare facilities), (11) company-provided emotional allowances (paid emotional exhaustion leave), (12) stress leave policies, (13) the creation of health protection manuals, (14) education using health protection manuals, and (15) posted signs prohibiting verbal harassment. Each respondent was asked to answer all questionnaire items.

To measure these factors, participants were selected from a binary “yes or no” for violent experiences. In addition, relevant follow-up systems implemented in the workplace included (1) the presence of systems to allow work interruptions and rest when workers are affected by verbal and physical violence, (2) Employment protections (3) systems for companies to provide support to employees filing lawsuits accusing customers of verbal harassment and demanding compensation, (3) support for treatment or counseling for health problems caused by verbally abusive customers, (4) the implementation of systems to restrict abusive customers from receiving service, and (5) systems to prohibit disadvantages for workers who request data needed to file lawsuits or make accusations.4) Data Analysis.

We analyzed the collected data using SPSS 23.0 (IBM Corp., Armonk, NY, USA). First, we analyzed participants’ general characteristics, occupational characteristics (i.e. A, B, and C), experiences of workplace violence, and protection systems in place for workers using the descriptive statistics method. Second, we analyzed general and occupational characteristics, experiences of workplace violence, and protection systems for workers using the chi-square test. Third, we examined how the protection system for workers affected workplace violence using the logistic regression method, with one item each for the general characteristics, occupational characteristics, and protection systems (significance level of *p* < 0.05 for the two-tailed test).

## Results

### General characteristics of participants

Table 1 shows general characteristics of the 1,349 total participants. Workplace violence was reported by 72.1% in sales, business, and call centers, 94.2% in electronic device repair, 84.6% with special employment status and visiting workers, and 79.7% in medicine, social welfare, and other fields. As for sex, 90.9% of male participants and 75.2% of female participants had experienced workplace violence; moreover, we found significant differences between these groups (*p*-value—= 0.001). As for age, workplace violence was reported by 71.0% of participants aged 29 years or below, 79.2% aged 40 to 49 years, and 82.9% aged 50 to 59 years; we found significant differences between these groups (*p*-value = 0.001). As for income, workplace violence was reported by 79.7% of participants with monthly incomes of two million KRW, 80.2% with monthly incomes of two to three million KRW, and 82.0% with monthly incomes of three million KRW or more. Finally, 85.3% of irregular workers and 78.6% of regular workers had experienced workplace violence; specifically looking at hours, 79.8% of those who worked 52 h per week or fewer and 88.5% of those who worked more than 52 h per week had experienced workplace violence.

**Table 1:** General characteristics, occupational characteristics, and workplace violence experiences across the study sample

Variable	Experiences of Workplace Violence		Total	χ^2^	p
	No	Yes			
Sex					
Male	43(9.1)	427(90.9)	470(100.0)	48.080	< .001
Female	218(24.8)	661(75.2)	879(100.0)		
Age (years)					
≤ 29	72(29.0)	176(71.0)	248(100.0)	25.836	< .001
30–39	92(20.8)	351(79.2)	443(100.0)		
40–49	54(13.3)	352(86.7)	406(100.0)		
50–59	43(17.1)	209(82.9)	252(100.0)		
Average Monthly Income					
< 2 million KRW	46(20.3)	181(79.7)	227(100.0)	0.642	.725
> 2 to < 3 million KRW	143(19.8)	580(80.2)	723(100.0)		
≥ 3 million KRW	72(18.0)	327(82.0)	399(100.0)		
Occupation Type					
Sales, business, and call centers	160(27.9)	414(72.1)	574(100.0)	65.236	< .001
Electronic device repair	18(5.8)	290(94.2)	308(100.0)		
Workers with special employment classifications and vising workers	37(15.4)	203(84.6)	240(100.0)		
Workers in medicine, social welfare, and other fields	46(20.3)	181(79.7)	227(100.0)		
Employment Type					
Irregular	61(14.7)	353(85.3)	414(100.0)	8.147	.004
Regular	200(21.4)	735(78.6)	935(100.0)		
Work Experience					
< 5 years	111(21.9)	397(78.1)	508(100.0)	3.287	.193
> 5 to < 10 years	61(17.6)	285(82.4)	346(100.0)		
> 10 years	89(18.0)	406(82.0)	495(100.0)		
Weekly Working Hours					
≤ 52 h	246(20.2)	973(79.8)	1219(100.0)	5.622	.019
> 52 h	15(15.4)	115(88.5)	130(100.0)		

### The relationship between experiences of workplace violence and the worker protection system

Table 2 presents information on the relationship between experiences of workplace violence and worker protection systems. As shown, we observed a 62.5% (*p* < 0.001) occurrence of workplace violence in businesses that provided service workers with regular counseling by professional counselors. This was lower compared to the 81.8% occurrence in businesses without such systems. We also found occurrences of 73% (*p* = 0.023) in businesses with departments or persons in charge of protecting service workers, and 74.9% (*p* = 0.040) in those with company policies to protect worker health. Both of these were lower than the occurrences in businesses without any such provisions. We observed occurrences of 68.1% (*p* = 0.007) in businesses that prepared separate budgets for service worker protection, and 71.7% (*p* = 0.007) in those conducting surveys to assess the state of emotional labor, types of customers, and health problems among workers. Both of these occurrences were lower than those found in businesses without any such practices. We found occurrences of 72.7% (*p* < 0.001) in businesses that authorized workers to discontinue services in response to excessive demands, and 74.8% (*p* = 0.009) in those with worker suggestion systems. Both occurrences were lower than those found in businesses without such systems. We observed occurrences of 72.7% (*p* < 0.001) in businesses with persons or departments in charge of grievance procedures, and 67.1% (*p* < 0.001) in those providing stress relief programs, both of which were lower than the occurrences found at workplaces without those systems. We found occurrences of 74% (*p* = 0.019) in businesses improving working conditions by installing rest and welfare facilities, 69.0% (*p* < 0.001) in those providing emotional allowances, and 69.7% (*p* < 0.001) in those offering emotional leave. All of these occurrences were lower than those found in businesses without such benefits. The occurrence of workplace violence in businesses providing health protection manuals was 82.7% (*p* = 0.001), and 82.9% (*p* < 0.001) in those offering education about health protection manuals. We observed occurrences of 81.8% (*p* = 0.024) in businesses with systems allowing work interruptions and rest when workers experienced verbal or physical abuse, and 69.8% (*p* = 0.002) in those prohibiting disadvantages for workers involved in disputes with clients. Both occurrences were lower than those found in workplaces without similar systems. Finally, we found occurrences of 72.8% (*p* = 0.049) in businesses supporting employees in filing lawsuits against clients accusing them of verbal abuse and demanding compensation, 72.8% (*p* = 0.003) in those assisting employees in receiving treatment for health problems or attending counseling, 74.5% (*p* = 0.039) in those implementing systems restricting clients who caused problems for workers, and 71.9% (*p* = 0.031) in those prohibiting any disadvantages for workers requesting data needed to file lawsuits and make accusations. All of these occurrences were lower than those found in businesses without such systems.

**Table 2:** The relationship between workplace violence experiences and worker protection systems

Variable	Experiences of Workplace Violence	Total	χ^2^	p			
	No	Yes					
Preliminary Actions	Regular counseling for service workers by professional counselors	No	231(18.2)	1038(81.8)	1269(100.0)	17.958	< .001
		Yes	30(37.5)	50(62.5)	80(100.0)		
	Designated department or person in charge of protecting service workers	No	227(18.6)	996(81.4)	1223(100.0)	5.194	.023
		Yes	34(27.0)	92(73.0)	126(100.0)		
	Company policies protecting service workers’ health	No	218(18.5)	960(81.5)	1178(100.0)	4.219	.040
		Yes	43(25.1)	128(74.9)	171(100.0)		
	Preparation of a separate budget for the protection of service workers	No	239(18.7)	1041(81.3)	1280(100.0)	7.324	.007
		Yes	22(31.9)	47(68.1)	69(100.0)		
	Surveys on the state of emotional labor, types of customers, and health problems of workers	No	225(18.4)	997(81.6)	1222(100.0)	7.276	.007
		Yes	36(28.3)	91(71.7)	127(100.0)		
	Authorization of workers to discontinue services in response to excessive demands (e.g., ending phone calls after a verbal warning)	No	189(17.4)	896(82.6)	1085(100.0)	13.211	< .001
		Yes	72(27.3)	192(72.7)	264(100.0)		
	Implementation of a worker suggestion system (e.g., online bulletin boards and grievance boxes)	No	198(18.0)	901(82.0)	1099(100.0)	6.735	.009
		Yes	63(25.2)	187(74.8)	250(100.0)		
	Designation of a person or committee in charge of grievance procedures	No	214(18.1)	968(81.9)	1182(100.0)	9.450	.002
		Yes	47(28.1)	120(71.9)	167(100.0)		
	Management of stress relief programs	No	215(17.8)	994(82.2)	1209(100.0)	18.270	< .001
		Yes	46(32.9)	94(67.1)	140(100.0)		
	Support for groups or clubs to enhance communication within the company	No	216(19.0)	921(81.0)	1137(100.0)	0.569	.451
		Yes	45(21.2)	167(78.8)	212(100.0)		
	Improvement of working conditions (e.g., the installation of rest and welfare facilities)	No	217(18.4)	963(81.6)	1180(100.0)	5.538	.019
		Yes	44(26.0)	125(74.0)	169(100.0)		
	Company-provided emotional allowances	No	131(14.1)	798(85.9)	929(100.0)	52.634	< .001
		Yes	130(31.0)	290(69.0)	420(100.0)		
	Company-provided emotional leave	No	155(15.5)	844(84.5)	999(100.0)	36.237	< .001
		Yes	106(30.3)	244(69.7)	350(100.0)		
	Company-created health protection manual	No	83(25.7)	240(74.3)	323(100.0)	10.970	.001
		Yes	178(17.3)	848(82.7)	1026(100.0)		
	Education related to a health protection manual	No	85(26.5)	236(73.5)	321(100.0)	13.731	< .001
		Yes	176(17.1)	852(82.9)	1028(100.0)		
	Posters and audio guides prohibiting verbal and physical abuse	No	107(21.6)	389(78.4)	496(100.0)	2.488	.116
		Yes	154(18.1)	699(81.9)	853(100.0)		
Follow-up Actions	Work interruptions and rest following verbal and/or physical abuse	No	59(24.8)	179(75.2)	238(100.0)	5.485	.024
		Yes	202(18.2)	909(81.8)	1111(100.0)		
	Prohibition of disadvantages for workers who had disputes with clients	No	226(18.3)	1007(81.7)	1233(100.0)	9.530	.002
		Yes	35(30.2)	81(69.8)	116(100.0)		
	Company support for employees filing lawsuits accusing clients of verbal abuse and demanding compensation	No	236(18.8)	1021(81.2)	1257(100.0)	3.875	.049
		Yes	25(27.2)	67(72.8)	92(100.0)		
	Support for treatment or counseling for health problems caused by verbal abuse	No	209(18.0)	949(82.0)	1158(100.0)	8.848	.003
		Yes	52(27.2)	139(72.8)	191(100.0)		
	Implementation of a system that restricts customers from causing problems (e.g., restriction of access)	No	221(18.5)	971(81.5)	1192(100.0)	4.279	.039
		Yes	40(25.5)	117(74.5)	157(100.0)		
	Prohibition of disadvantages for workers Employment protections when they request data needed to file lawsuits and make accusations	No	236(18.7)	1024(81.3)	1260(100.0)	4.667	.031
		Yes	25(28.1)	64(71.9)	89(100.0)		

### Correlation analysis between protection system and experience of violence.

Table 3 shows Pearson's correlation analysis was conducted to confirm the correlation between protection systems, which are the variables of this study. As a result, the variables showed significant correlation.

**Table 3:** Correlation analysis between protection system and experience of violence

	1	2	3	4	5	6	7	8	9	10	11	12	13	14	15	16	17	18	19	20	21	22
1	1																					
2	.361^**^	1																				
3	.376^**^	.419^**^	1																			
4	.427^**^	.399^**^	.437^**^	1																		
5	.436^**^	.419^**^	.517^**^	.422^**^	1																	
6	.336^**^	.348^**^	.423^**^	.276^**^	.373^**^	1																
7	.317^**^	.324^**^	.345^**^	.288^**^	.396^**^	.347^**^	1															
8	.439^**^	.372^**^	.369^**^	.342^**^	.395^**^	.314^**^	.497^**^	1														
9	.457^**^	.321^**^	.334^**^	.338^**^	.387^**^	.338^**^	.292^**^	.348^**^	1													
10	.296^**^	.196^**^	.238^**^	.214^**^	.258^**^	.208^**^	.270^**^	.355^**^	.389^**^	1												
11	.358^**^	.337^**^	.324^**^	.327^**^	.360^**^	.269^**^	.410^**^	.459^**^	.350^**^	.380^**^	1											
12	.172^**^	.202^**^	.278^**^	.187^**^	.186^**^	.355^**^	.144^**^	.104^**^	.174^**^	.063^*^	.132^**^	1										
13	.203^**^	.234^**^	.303^**^	.217^**^	.228^**^	.361^**^	.160^**^	.148^**^	.185^**^	.099^**^	.128^**^	.835^**^	1									
14	-.134^**^	-.168^**^	-.231^**^	-.122^**^	-.165^**^	-.204^**^	-.131^**^	-.149^**^	-.176^**^	-.149^**^	-.097^**^	-.094^**^	-.106^**^	1								
15	-.175^**^	-.167^**^	-.280^**^	-.130^**^	-.187^**^	-.192^**^	-.153^**^	-.178^**^	-.184^**^	-.191^**^	-.143^**^	-.112^**^	-.144^**^	.570^**^	1							
16	-.112^**^	-.158^**^	-.227^**^	-.110^**^	-.185^**^	-.240^**^	-.149^**^	-.094^**^	-.165^**^	-.150^**^	-.101^**^	-.235^**^	-.237^**^	.382^**^	.351^**^	1						
17	-.154^**^	-.119^**^	-.189^**^	-.151^**^	-.178^**^	-.173^**^	-.127^**^	-.166^**^	-.143^**^	-.161^**^	-.151^**^	-0.006	-0.030	.333^**^	.340^**^	.285^**^	1					
18	.314^**^	.434^**^	.492^**^	.420^**^	.470^**^	.334^**^	.342^**^	.356^**^	.291^**^	.272^**^	.336^**^	.176^**^	.210^**^	-.115^**^	-.189^**^	-.180^**^	-.133^**^	1				
19	.369^**^	.468^**^	.365^**^	.459^**^	.357^**^	.283^**^	.303^**^	.327^**^	.292^**^	.223^**^	.305^**^	.214^**^	.237^**^	-.114^**^	-.122^**^	-.110^**^	-.115^**^	.544^**^	1			
20	.412^**^	.395^**^	.421^**^	.332^**^	.424^**^	.375^**^	.274^**^	.351^**^	.382^**^	.350^**^	.302^**^	.240^**^	.276^**^	-.177^**^	-.183^**^	-.206^**^	-.217^**^	.443^**^	.381^**^	1		
21	.282^**^	.407^**^	.411^**^	.305^**^	.399^**^	.382^**^	.275^**^	.285^**^	.314^**^	.219^**^	.302^**^	.168^**^	.162^**^	-.178^**^	-.188^**^	-.206^**^	-.211^**^	.424^**^	.362^**^	.418^**^	1	
22	.384^**^	.471^**^	.448^**^	.448^**^	.420^**^	.308^**^	.321^**^	.383^**^	.314^**^	.241^**^	.334^**^	.147^**^	.173^**^	-.146^**^	-.175^**^	-.142^**^	-.152^**^	.632^**^	.585^**^	.409^**^	.420^**^	1

1. Regular counseling for service workers by professional counselors

2. Designated department or person in charge of protecting service workers

3. Company policies protecting service workers’ health

4. Preparation of a separate budget for the protection of service workers

5. Surveys on the state of emotional labor, types of customers, and health problems of workers

6. Authorization of workers to discontinue services in response to excessive demands

7. Implementation of a worker suggestion system

8. Designation of a person or committee in charge of grievance procedures

9. Management of stress relief programs

10. Support for groups or clubs to enhance communication within the company

11. Improvement of working conditions

12. Company-provided emotional allowances

13. Company-provided emotional leave

14. Company-created health protection manual

15. Education related to a health protection manual

16. Posters and audio guides prohibiting verbal and physical abuse

17. Work interruptions and rest following verbal and/or physical abuse

18. Prohibition of disadvantages for workers who had disputes with clients

19. Company support for employees filing lawsuits accusing clients of verbal abuse and demanding compensation

20. Support for treatment or counseling for health problems caused by verbal abuse

21. Implementation of a system that restricts customers from causing problems

22. Prohibition of disadvantages for workers Employment protections when they request data needed to file lawsuits and make accusations

### The effect on experiences of violence according to the protection system

Table 4 shows how specific protection systems affected experiences of workplace violence. We jointly analyzed the service worker protection system and general/occupational characteristics of participants. As such, we found that the likelihood of experiencing workplace violence at businesses that provided regular counseling by professional counselors was 0.46 times lower (95% confidence interval [CI] = 0.277–0.748) than at those where counseling was not provided. The same likelihood was 0.67 times lower at businesses that had persons or departments in charge of grievance procedures (95% CI = 0.45–0.99) than at those which did not. Finally, the likelihood of workplace violence was 0.56 times lower at businesses with stress relief programs (95% CI = 0.373–0.836) than at those without.

**Table 4:** Effects on experiences of violence according to the protection system

Action	Variable	OR	95% CI		p	χ^2^*	R^2^
			Lower	Upper			
Preliminary Actions	Regular counseling for service workers by professional counselors	0.46	0.28	0.75	.002	3.372	.111
	Designated department or person in charge of protecting service workers	0.84	0.54	1.29	.419	3.119	.101
	Company policies protecting service workers’ health	0.98	0.66	1.45	.924	3.116	.101
	Preparation of a separate budget for the protection of service workers	0.63	0.36	1.08	.093	4.136	.104
	Surveys on the state of emotional labor, types of customers, and health problems of workers	0.78	0.51	1.20	.266	4.460	.102
	Authorization of workers to discontinue services in response to excessive demands (e.g., ending phone calls after a verbal warning)	0.84	0.60	1.19	.327	3.602	.102
	Implementation of a worker suggestion system (e.g., online bulletin boards and grievance boxes)	0.85	0.60	1.19	.342	3.476	.102
	Designation of a person or committee in charge of grievance procedures	0.67	0.45	0.99	.045	2.159	.105
	Management of stress relief programs	0.56	0.37	0.84	.005	4.124	.109
	Support for groups or clubs to enhance communication within the company	0.81	0.54	1.19	.276	6.581	.102
	Improvement of working conditions (e.g., the installation of rest and welfare facilities)	0.84	0.57	1.24	.382	4.011	.102
	Company-provided emotional allowances	0.66	0.42	1.04	.074	12.088	.104
	Company-provided emotional leave	0.82	0.57	1.20	.311	7.992	.102
	Company-created health protection manual	1.13	0.82	1.56	.451	10.522	.101
	Education related to a health protection manual	1.21	0.83	1.66	.236	5.189	.102
	Posters and audio guides prohibiting verbal and physical abuse	0.85	0.63	1.15	.299	2.738	.102
Follow-up Actions	Work interruptions and rest following verbal and physical abuse	1.04	0.73	1.47	.839	3.528	.101
	Employment protections who had disputes with clients	0.67	0.43	1.03	.070	3.014	.104
	Company support for employees filing lawsuits accusing clients of verbal abuse and demanding compensation	0.86	0.52	1.41	.546	3.903	.101
	Support for treatment or counseling for health problems caused by verbal abuse	0.77	0.53	1.12	.168	5.757	.103
	Implementation of a system that restricts customers from causing problems (e.g., restriction of access)	0.89	0.60	1.32	.557	5.081	.101
	Employment protections when they request data needed to file lawsuits and make accusations	0.75	0.45	1.23	.252	3.510	.102

*CI* Confidence interval, *OR* Odds ratio

^*^Adjusted for sex, age category, occupational type, employment type, and weekly working hours; these were applied as control variables for each of the service worker protection systems

^*^χ^2^: Hosmer & Lemeshow

## Discussion

Focusing on service workers, this study investigated the relationships between general personal characteristics, occupational characteristics, experiences of workplace violence, and the existence of an employee protection system. We then estimated whether the adoption of that protection system had reduced workplace violence. Our primary conclusions are organized into two subsections:

1. Relationships between the general/occupational characteristics of service workers and workplace violence

First, workplace violence were higher among females, older workers, electronic equipment repairers, irregular workers, and individuals who had worked for extended periods. These findings support the results of some previous studies, indicating that workplace violence is more occurred among females than males [23], which may be due to the tendency for male workers to have a lower threshold and tolerance for unreasonable demands and abusive language and actions from clients [24]. However, there were also contrasting findings. For example, one study found that older medical personnel had a lower risk of workplace violence than their colleagues and other workers due to their accumulation of experience with nursing patients [25], while this study found that older workers experienced more workplace violence compared to younger workers. This may be because older workers are more likely to be in decision-making positions and therefore address customer complaints more frequently [26].

We also found that experiences of workplace violence varied according to occupation type. Of note, electronic device repairers were particularly vulnerable to such occurrences. As these workers are typically given positive reviews only when customers are satisfied with their work, they are more dependent on client satisfaction than others. In turn, they may have more negative outcomes and fewer avenues for recourse when their businesses do not have policies that protect them from workplace violence, especially when they are not given direct support during such instances [27].

Moreover, precariousness at work is associated with workplace violence [28, 29]. For example, irregular workers are more likely to be excluded from various benefits and institutional support systems [30]. This often creates feelings of anxiety, alienation, and frustration, all of which make it more difficult to communicate with customers [31], thus increasing the likelihood of workplace violence [32, 33].

Finally, we found that participants who worked more than 52 h per week have experienced more workplace violence, which supports existing evidence from Korea, Japan, and China showing a higher risk of such violence among individuals who work for extended periods [34]. In this regard, workplace violence is a sociopsychological hazard that may be more likely to occur the longer a worker remains on duty [29]. Meanwhile, working for extended periods is known to cause fatigue and anger, which can further increase the risk of such violence [29, 35].

2) Relationship between a protection system for service workers and experiences of workplace violence

Of the preliminary actions and protection systems for service workers, we found lower rates of workplace violence in cases where regular counseling was provided by professional counselors. Indeed, several studies have shown that counseling is an essential aspect of responding to workplace violence [36, 37]. As a form of counseling, it is also important to provide mentoring programs that allow experienced professionals to teach and guide individuals with less experience, thus establishing a culture of interpersonal care and respect [38].

As mentioned earlier, we found a low rate of workplace violence at businesses with persons or departments in charge of grievance procedures. Although we cannot examine this in greater comparative detail because no such systems were examined in previous studies, our findings partially correspond to those reported by Lee et al. [29]. In this study, protection systems, violence management, and health guidelines were crucial for organizations, while health risks increased among workers in locations where these support systems were not established. We also found lower rates of workplace violence in businesses with stress relief programs. In this regard, workplaces that provide psychosocial and support services [6] to both managers and workers are more likely to have low rates of such violence, as these systems are well-equipped overall. While relief programs are effective, Kwak, Han [37] further argued for the need to develop skills and behavior management education programs aimed at violence management, including direct interventions in attack situations. These programs should include materials on communication techniques, the psychology of service to help understand customer demands, and methods for properly responding to those demands [39]. Moreover, businesses should offer violence prevention programs and team-building workshops to help alleviate the effects of parallel violence, since these programs are known to reduce the risk of negative confrontations with aggressive customers [17]. Other sequential programs can also establish positive cultures for conflict management [3] and mutual respect in the workplace [40].

At the 108^th^ International Labour Organization (ILO) convention on June 10, 2019, members around the world were encouraged to prevent violence and harassment, especially by developing policies that recognize the importance of workplace culture and prevent workplace violence based on human dignity [6]. Thus, ILO members are preparing policies to meet these demands, which should facilitate future agreements and systems that reduce violence and harassment in workplaces across the globe. While Italy was the first European nation to implement a service worker protection system (January 15, 2021) [41], this was preceded by the system implemented in South Korea (October 28, 2018). The current study is distinct from others on workplace violence because few other independent worker protection systems exist, although they have been discussed in the context of civil laws and codes in some countries. Thus, our research yielded particularly meaningful insights due to Korea’s unique, detailed, and well-established protection system for service workers.

This study also had some limitations. First, we used a cross-sectional approach to examine a service worker protection system after it was created, with a sole focus on the correlation between that system and experiences of workplace violence. Second, we only investigated conditions among service workers, meaning that our results cannot directly be generalized to the overall population. Third, we used crude odds ratios to measure associations, which does not control for potential confounding variables. This implies that the observed relationships between workplace violence and the studied factors could be influenced by unaccounted variables. For instance, age, gender, socio-economic status, and prior experiences of violence are factors that might influence the likelihood of experiencing workplace violence. Therefore, the relationships we found should be interpreted with caution as the true relationships could be over- or under-estimated due to these potential confounders. Furthermore, our analysis does not account for the hierarchical nature of the data. This refers to the potential clustering of our data that might exist, for example, within organizations or within certain professional groups. Ignoring such hierarchical structure can lead to an underestimation of standard errors, and thus overstate the statistical significance of predictors. Our results, therefore, may not adequately reflect variations within these potential clusters and this limits the generalizability of our findings. These limitations have important implications for the validity of our results. While our study provides a useful starting point in exploring workplace violence, it is crucial for future research to conduct more nuanced analyses. More sophisticated statistical techniques, such as multilevel modeling and calculation of adjusted odds ratios, should be employed to control for confounding variables and account for the hierarchical nature of the data. This would allow for a more accurate and comprehensive understanding of the factors associated with workplace violence. Last, our study design did not employ multilevel regression, which would have been more appropriate for assessing the influence of an organizational-level factor (workplace protection system) on an individual-level factor (worker's experience of workplace violence). This methodological choice may have limited the accuracy and generalizability of our findings. Future research should consider utilizing multilevel regression to better understand the impact of organizational factors on individual experiences of workplace violence.

Despite this, our study makes valuable contributions to the literature. First, our results show that service worker protection systems are effective. This is important information for countries where systems are not yet established and/or where business owners are not required to comply. Second, we found that active worker engagement influenced the development of a workplace culture in which violence was prevented; at the same time, appropriate organizational management enhanced these effects. Future studies should add to this by investigating individual forms of workplace violence as well as the long-term effects of worker protection systems. In Korea, this will become comparatively more observable over time, as our research was conducted only two years after system implementation.

## Conclusions

The current findings constitute a basis for expanding Korea’s existing worker protection system by clarifying the correlation between the adoption of the protection system for service workers and the occurrence of workplace violence. We also made empirical observations demonstrating the protection system’s effectiveness. However, the system has only been implemented for a short time, which precludes any discussions on its long-term effects, thus highlighting the need for continued research. At this time, we recommend that workplaces either designate individuals to oversee grievance procedures or establish grievance procedure committees, as both measures effectively reduced workplace violence in this study. Further, businesses should work to reduce the effects of workplace violence on worker health by providing regular counseling with professional counselors. Such effects may become clearer after implementing stress relief programs to reduce stress and minimize any psychological impacts. Meanwhile, Korean government agencies should continuously monitor the effects of the protection system, especially to determine whether workplaces are complying with the preliminary and follow-up actions stipulated in the Occupational Safety and Health Act.

## Data Availability

The datasets used and/or analysed during the current study available from the corresponding author on reasonable request.

## Abbreviation

ILO: International Labour Organization
